# The Neuroprotective Properties of *Hericium erinaceus* in Glutamate-Damaged Differentiated PC12 Cells and an Alzheimer’s Disease Mouse Model

**DOI:** 10.3390/ijms17111810

**Published:** 2016-11-01

**Authors:** Junrong Zhang, Shengshu An, Wenji Hu, Meiyu Teng, Xue Wang, Yidi Qu, Yang Liu, Ye Yuan, Di Wang

**Affiliations:** 1 School of Life Sciences, Jilin University, Changchun 130012, China; archer707@163.com (J.Z.); anshengshu@163.com (S.A.); huwj1312@163.com (W.H.); tengmeiyu2013@163.com (M.T.); xuew16@mials.jlu.edu.cn (X.W.); yidiqu@163.com (Y.Q.); liuyangyang@jlu.edu.cn (Y.L.); 2 Department of Laboratory Medicine, The First Hospital of Jilin University, Changchun 130021, China; papas93@163.com

**Keywords:** *Hericium erinaceus*, neuro-protection, glutamate, mitochondria, Alzheimer‘s disease mouse

## Abstract

*Hericium erinaceus*, an edible and medicinal mushroom, displays various pharmacological activities in the prevention of dementia in conditions such as Parkinson’s and Alzheimer’s disease. The present study explored the neuroprotective effects of *H. erinaceus* mycelium polysaccharide-enriched aqueous extract (HE) on an l-glutamic acid (l-Glu)-induced differentiated PC12 (DPC12) cellular apoptosis model and an AlCl_3_ combined with d-galactose-induced Alzheimer’s disease mouse model. The data revealed that HE successfully induced PC12 cell differentiation. A 3 h HE incubation at doses of 50 and 100 µg/mL before 25 mM of l-Glu effectively reversed the reduction of cell viability and the enhancement of the nuclear apoptosis rate in DPC12 cells. Compared with l-Glu-damaged cells, in PC12 cells, HE suppressed intracellular reactive oxygen species accumulation, blocked Ca^2+^ overload and prevented mitochondrial membrane potential (MMP) depolarization. In the Alzheimer’s disease mouse model, HE administration enhanced the horizontal and vertical movements in the autonomic activity test, improved the endurance time in the rotarod test, and decreased the escape latency time in the water maze test. It also improved the central cholinergic system function in the Alzheimer’s mice, demonstrated by the fact that it dose-dependently enhanced the acetylcholine (Ach) and choline acetyltransferase (ChAT) concentrations in both the serum and the hypothalamus. Our findings provide experimental evidence that HE may provide neuroprotective candidates for treating or preventing neurodegenerative diseases.

## 1. Introduction

Chronic progressive degenerative damage to the central nervous system can cause neurodegenerative diseases such as Alzheimer’s disease (AD) and Parkinson’s disease (PD), both of which have drawn the attention of researchers due to their devastating nature and limited therapeutic options [1]. AD, an irreversible degenerative disease and the main cause of dementia, affects 60%–65% of people worldwide and frequently occurs among the elderly [2]. The main clinical manifestations of AD are cognitive function decline and a poor prognosis, and patients generally die of pulmonary embolism or a secondary infection [3]. Although the pathomechanisms of neurodegenerative diseases remain largely unknown, hypotheses featuring neuronal apoptosis has emerged as an attractive theory supported by observations of apoptotic cells in affected brain regions [4]. During the development process for AD, neurons exhibit pathological features, especially in relation to mitochondrial dysfunction [5]. In the mitochondria-mediated apoptotic process, the overload of intracellular calcium produces large amounts of reactive oxygen species (ROS) [6], which promotes the opening of mitochondrial permeability transition pores (mPTPs), prompting the dissipation of mitochondrial membrane potential (MMP) [7]. Furthermore, functional and structural damage induced by AD in brain tissues results in energy deficiency, increased ROS, and neuronal death [8].

For in vitro studies on neurodegenerative diseases, rat pheochromocytoma cells (PC12 cells) are recognized as a common model because they display obvious synapse formation and are capable of producing nerve-related proteins, such as neurons [9]. As an important excitatory neurotransmitter in the central nervous system, dramatically enhanced glutamate levels generate excitatory neurotoxicity [10]. Synaptic cleft glutamate levels regulated by nerve and glial cells cannot accumulate to a toxic concentration; however, in pathological conditions, excessive activated extracellular glutamate receptors (particularly the ionotropic receptors of *N*-methyl-d-aspartate (NMDA)) prompt changes in ion channels and signaling systems. This ultimately leads to neuronal death [11]. In this study, the AD mouse model was established by combining a d-galactose (d-gal) injection with AlCl_3_ intragastric administration, which induced AD-like behavior and readily produced pathological alterations, unlike those treated with either AlCl_3_ or d-gal exclusively [12].

Thus far there have been no satisfactory therapeutic options for AD patients. Due to patients’ various biological responses, herbs and fungi have been applied as medicine or functional food for centuries, and as such have become a repository for biopharmaceutic development [1]. According to previous research, numerous polysaccharides extracted from fungus display neuroprotective properties [13]. Our group successfully isolated a polysaccharide from *Sparassis crispa* that protects DPC12 cells against l-glutamic acid (l-Glu)-induced apoptosis via the mitochondrial apoptotic pathway [14]. *Hericium erinaceus*, an edible and medicinal mushroom, displays various pharmacological activities in the prevention of dementia in conditions such as PD and AD [15]. *H. erinaceus* water extracts have been reported to show antitumor, antimutagenic, antioxidant, hypolipidemic, and immunomodulatory properties in cell and animal models [16]. Encouragingly, *H. erinaceus* displays neuroprotective properties, such as facilitating nerve growth factor (NGF) expression and secretion and regulating the differentiation and development of cholinergic neurons in in vitro and in vivo experiments [2]. Recently, *H. erinaceus* has been reported to improve visual cognitive memory and mediate spatial short-term memory damage in an Aβ-induced mouse model analyzed via behavioral tests [17]. Taken together, these studies suggest that *H. erinaceus* may have beneficial effects on neurodegenerative diseases.

In the present study, the neuroprotection of an *H. erinaceus* polysaccharide-enriched aqueous extract (HE) was investigated in an l-Glu-induced DPC12 cellular apoptosis model and an AlCl_3_ combined with d-gal-induced AD mouse model. Our data revealed that HE improved cell viability and reversed nuclear apoptotic alternation via the mitochondrial related pathway. The experiments performed in the AD mouse model further confirmed the therapeutic effects of HE on AD, which provided helpful evidence of the benefits of using *H. erinaceus* as a medicine or functional food application in neurodegenerative diseases.

## 2. Results

### 2.1. HE-Induced PC12 Cell Differentiation

**Figure 1:**
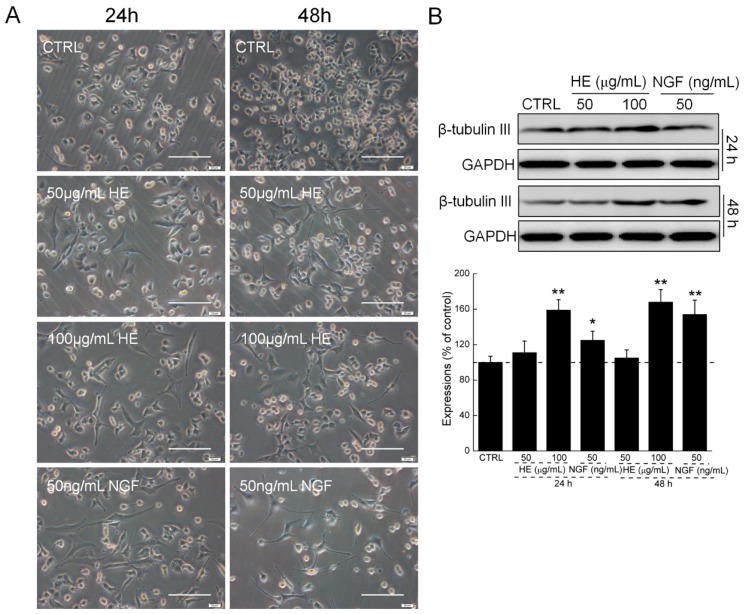
(**A**) HE and NGF induced PC12 cell differentiation determined by inverted microscope. Scale bar: 100 µm. (*n* = 6); (**B**) HE and NGF enhanced the expressions of β-tubulin III in PC12 cells. Data are expressed as mean ± SD (*n* = 6). * *p* < 0.05 and ** *p* < 0.01 vs. CTRL, HE: *Hericium erinaceus* aqueous extract; NGF: Nerve growth factor; CTRL: Non-treated cells.

Comparatively, the control cell types were round, triangular, and irregular short spindle, and exhibited well refraction under an inverted microscope. Similarly, regarding NGF, after incubation with 50 and 100 µg/mL of HE for 24 and 48 h, respectively, the bodies of the PC12 cells became polygons or polygonal. Combined with the increased HE concentration, the cell axons were similar to neurons, and the differentiation rate was significantly enhanced (Figure 1A). Both NGF and HE strongly enhanced the expressions of β-tubulin III in PC12 cells after 24 and 48 h incubation (Figure 1B).

### 2.2. HE Improved Cell Viability and Nuclear Apoptotic Alternation

**Figure 2:**
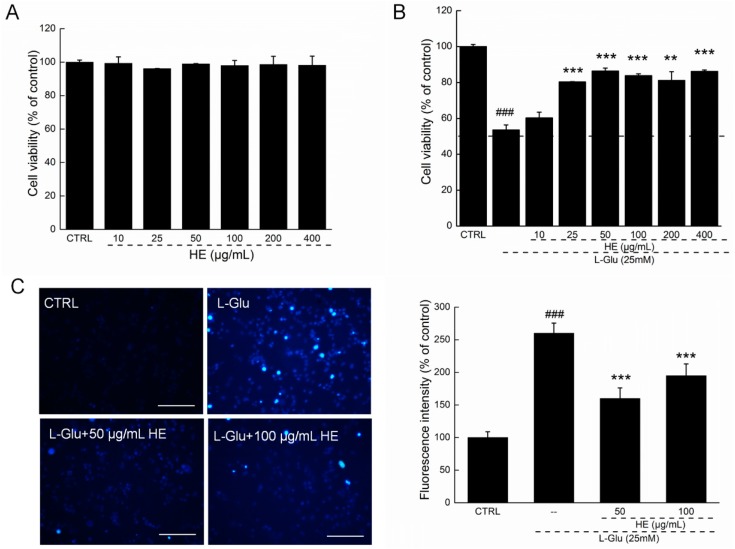
(**A**) HE showed no effects on cell proliferation in normal DPC12 cells; (**B**) but strongly enhanced cell viability in l-Glu-induced DPC12 cellular apoptosis model; (**C**) HE restored l-Glu-induced nucleus morphological apoptotic alterations analyzed via Hoechst 33342 staining (*n* = 6). Scale bar: 100 µm. HE: *Hericium erinaceus* aqueous extract. Data are expressed as mean ± SD (*n* = 6). ^###^ *p* < 0.001 vs. CTRL, ** *p* < 0.01 and *** *p* < 0.001 vs. l-Glu-exposed cells. HE: *Hericium erinaceus* aqueous extract; CTRL: Non-treated cells.

In the MTT assay, HE alone had no effect on DPC12 cell proliferation (Figure 2A). Twenty-four-hour exposure to 25 mM of l-Glu resulted in a 46.4% reduction in cell viability (*p* < 0.001; Figure 2B). Meanwhile, 3 h HE preincubation improved cell vitality by 30%, compared with l-Glu-exposed DPC12 cells (*p* < 0.01; Figure 2B).

Under Hoechst 33342 staining, the nontreated cells exhibited homogeneous staining with regular contours and rounded shapes. l-Glu caused asymmetric, bright-blue fluorescence patterns in cells that were significantly prevented by HE pretreatment at doses of 50 and 100 µg/mL, indicating HE’s ability to successfully inhibit nuclear apoptosis (*p* < 0.001; Figure 2C).

### 2.3. HE Reversed Mitochondrial Dysfunction, Ca^2+^ Overload and ROS Accumulation

**Figure 3:**
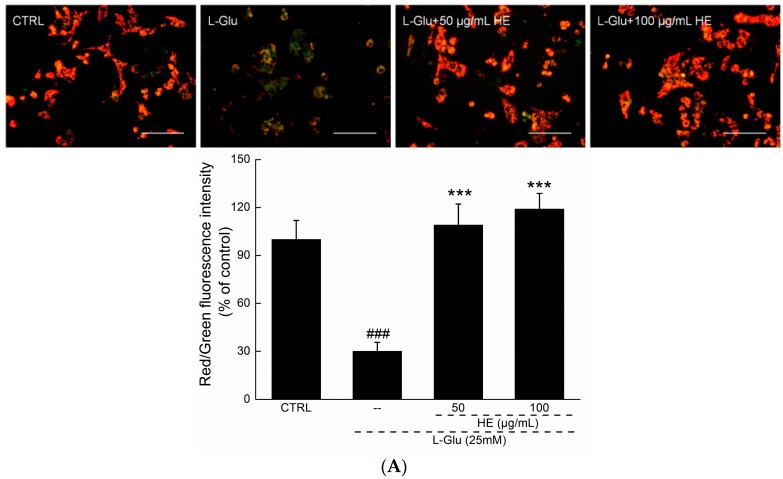

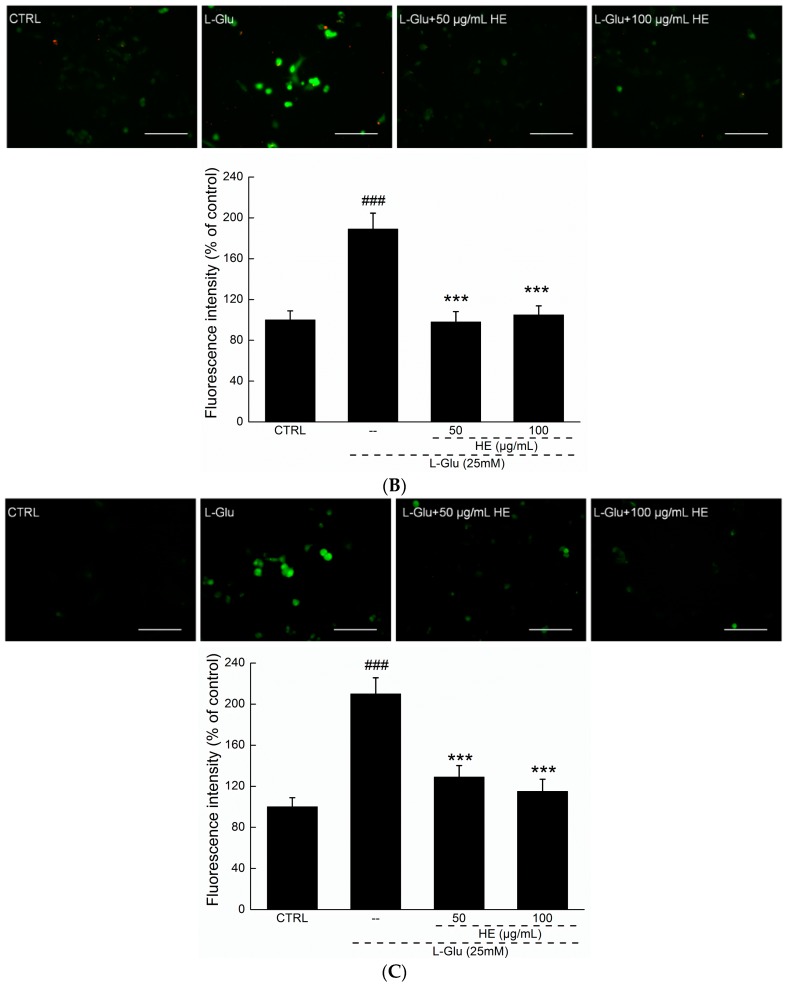
(**A**) The disruption of mitochondrial membrane potential (MMP) caused by 12 h l-Glu exposure was strongly restored by 3 h HE pretreatment analyzed via JC-1 staining (*n* = 6). Scale bar: 100 µm; (**B**) the overaccumulation of reactive oxygen species (ROS) caused by 12 h l-Glu exposure was significantly reduced by 3 h HE pretreatment analyzed by DCFH-DA (2’,7’-dichlorofluorescein diacetate) staining (*n* = 6). Scale bar: 100 µm; (**C**) intracellular Ca^2+^ overload caused by l-Glu was strongly reversed by 3 h HE pretreatment analyzed via Fluo-4-AM staining (*n* = 6). Scale bar: 100 µm. Data are expressed as mean ± SD (*n* = 6). ^###^ *p* < 0.001 vs. CTRL, *** *p* < 0.001 vs. l-Glu-exposed cells. HE: *Hericium erinaceus* aqueous extract; CTRL: Non-treated cells.

JC-1 (5,5’,6,6’-tetrachloro-1,1’,3,3’-tetraethylbenzimidazolylcarbocyanine iodide) molecular probe staining was applied to detect mitochondrial function. Although the control cells exhibited the intense red fluorescence indicative of healthy cells, the 12 h l-Glu-incubated cells exhibited the intense green fluorescence indicative of mitochondrial apoptosis (*p* < 0.001; Figure 3A). Comparatively, HE preincubation strongly enhanced the ratio of red-to-green fluorescence, suggesting its beneficial activity on mitochondria (*p* < 0.001; Figure 3A).

Three-hour HE preincubation followed by another 12 h coexposure to l-Glu strongly reduced high green fluorescence in DCFH-DA (2’,7’-dichlorofluorescein diacetate) staining, suggesting its inhibition of ROS accumulation (*p* < 0.001; Figure 3B), which was also confirmed using the flow cytometry method (Figure S1).

The application of 25 mM of l-Glu resulted in calcium overload in DPC12 cells after a 12 h exposure, as indicated by an extremely high green fluorescence (*p* < 0.001; Figure 3C). HE (50 and 100 µg/mL) significantly reduced green fluorescence, revealing its suppressive effect on intracellular calcium overload (*p* < 0.001; Figure 3C).

### 2.4. The Effects of HE on the Behavior of AD Mice

**Figure 4:**
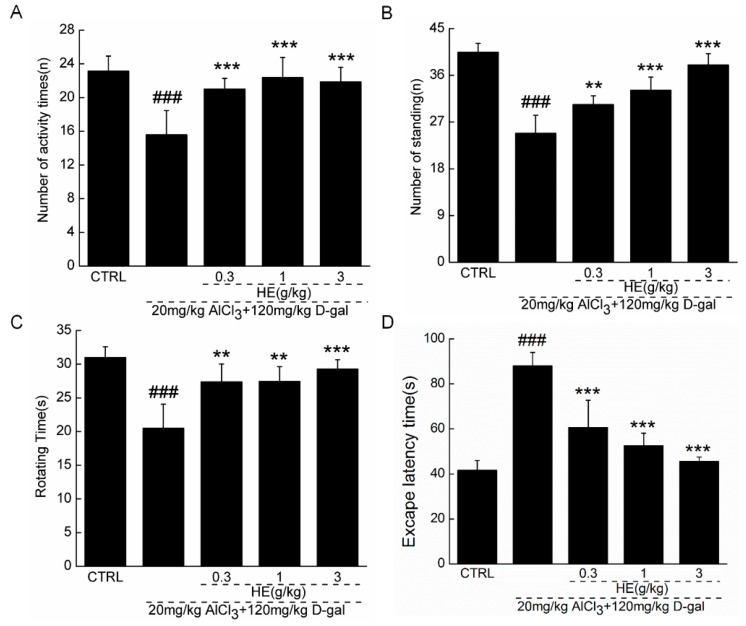
In AlCl_3_- and d-gal-induced AD mice, four-week HE treatment strongly enhanced (**A**) horizontal movements and (**B**) vertical movements in locomotor activity test; (**C**) increased the endurance time in rotating test; and (**D**) reduced the escape latency time in water-maze test compared with nontreated model mice. Data are expressed as mean ± SD (*n* = 10). ^###^ *p* < 0.001 vs. normal mice (CTRL), ** *p* < 0.01 and *** *p* < 0.001 vs. AD mice. HE: *Hericium erinaceus* aqueous extract.

The application of 0.3–1.0 g/kg of HE had no significant effect on the bodyweight of AD mice during the four-week administration period (Table 1). To further confirm HE’s beneficial activity on AD, the model mice were subjected to behavioral testing. In an autonomic activity test, HE strongly increased the horizontal and vertical movements of AD mice (*p* < 0.01; Figure 4A,B). Compared with the nontreated model mice, HE enhanced endurance time in the rotarod test by 30% (*p* < 0.05; Figure 4C). Importantly, to evaluate the effects of HE on learning and memory, we conducted a water maze test because it is commonly used to inspect the cognitive abilities of animals [18]. In AD mice, escape latency time was doubled compared with normal mice (*p* < 0.001; Figure 4D). Four-weeks of HE administration also resulted in a significant decrease in escape latency times (*p* < 0.001; Figure 4D).

### 2.5. HE Regulated Ach and ChAT Concentrations in Serum and Hypothalamus

**Figure 5:**
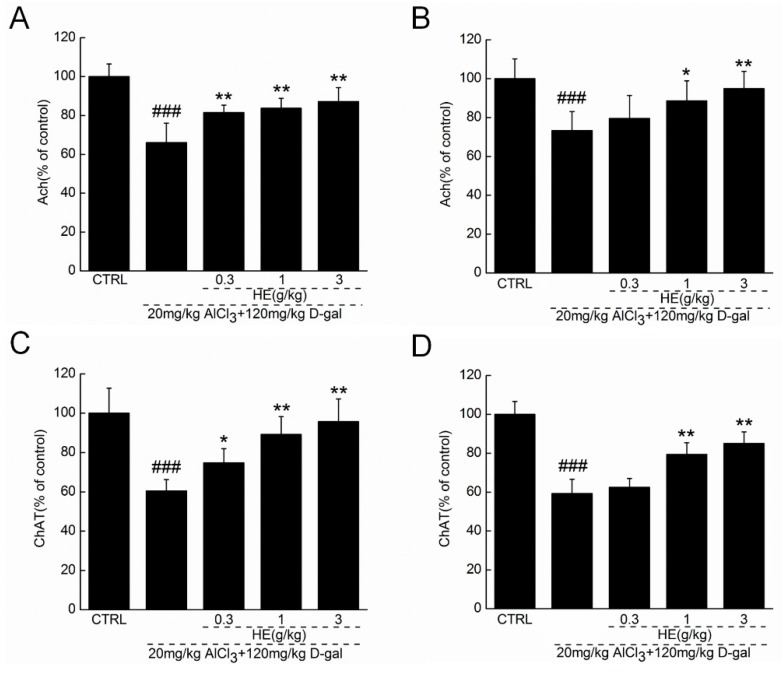
HE treated AlCl_3_- and d-gal-induced AD mice for four weeks, and blood and hypothalamus were collected. The levels of (**A**) acetylcholine (Ach) and (**C**) choline acetyltransferase (ChAT) in hypothalamus, and the levels of (**B**) Ach and (**D**) ChAT in serum were detected via ELISA method. Data are expressed as the percentage to controls and mean ± SD (*n* = 10). ^###^ *p* < 0.001 vs. normal mice (CTRL), * *p* <0.05 and ** *p* < 0.01 vs. AD mice. HE: *Hericium erinaceus* aqueous extract.

Extremely low acetylcholine (Ach) and choline acetyltransferase (ChAT) concentrations in serum and the hypothalamus were noted in the AlCl_3_- and d-gal-induced AD mice (*p* < 0.001; Figure 5), suggesting that the model successfully copied the central cholinergic function in the AD obstacle condition. Encouragingly, HE improved the central cholinergic system function of AD mice, as demonstrated by its dose-dependent enhancement of Ach and ChAT concentrations in both serum and the hypothalamus (*p* < 0.05; Figure 5).

## 3. Discussion

Consistent with previous studies [13], the present research clearly confirmed the neuroprotective effects of HE in cells and an AD mouse model, as demonstrated by a decrease in cell viability caused by strongly ameliorated neurotoxin, nuclear and mitochondrial apoptotic alternations, and abnormal behavior in mice. To our knowledge, this is the first confirmation of the neuroprotective effects of HE in l-Glu-induced DPC12 apoptotic cells, and a chemical drug (d-gal and AlCl_3_)-induced AD mouse model.

Unlike other AD treatment drugs or candidates, HE is an extraction from a fungus containing multiple active ingredients that appear to “systemically target” and eliminate the symptoms of disease in a surprisingly natural way. As an Asian folk remedy/functional food, the safety of *H. erinaceus* has been verified as having few side effects over centuries of use. Given that macrofungi grow on liquid culture, the fruiting body’s growth cycle is too long to complete, which makes product quality control difficult. These days, mycelia obtained from submerged fermentation have been used as substitutes for wild fruit bodies [19]. Our preliminary data show that *H. erinaceus* mycelium and fruit bodies contain similar active ingredients, including polysaccharides, fatty acids, and amino acids. In our acute toxicity test, HE failed to influence the daily performance and behavior of mice, which confirmed its safety. In contrast, the crude nature of HE suggests multi-effective components, which may explain its non-dose-dependent manner in some of our present data. The amount of natural productions is reported to show pharmacological activities and a non-dose-dependent response, which is considered common among natural drugs [20,21].

For the in vitro study, 25 mM of l-Glu was used to produce an apoptotic DPC12 cell model, which is consistent with previous research [22,23]. Our previous study on the neuroprotection of *S. crispa* provided experimental evidence supporting the use of 25 mM of l-Glu for model development [14]. The robust neuroprotective effect of HE is associated with its suppression of ROS accumulation and the Ca^2+^ overload caused by l-Glu. Free radical accumulation is an essential factor contributing to cellular apoptosis, and numerous natural products have been shown to control intracellular free radical levels [24]. HE has also been reported to display strong antioxidant effects, which may be directly associated with its broad therapeutic benefits [16]. As a hub for Ca^2+^ signaling, which is an effector of ATP, mitochondria are considered central to intracellular energy metabolism [25]. Interestingly, intracellular Ca^2+^ overload caused the opening of mPTPs and free radical accumulation, especially ROS, which led to mitochondrial depolarization and proapoptotic cytokine release [26]. Moreover, ROS accumulation exhibited short-loop feedback with mitochondrial dysfunction. The overproduction of ROS prompted the dissipation of MMP, which further increased ROS release from the mitochondria to the cytoplasm [27]. Both previous findings and our present data suggest that mitochondrial apoptotic signaling plays a central role in HE-mediated neuroprotection against l-Glu-induced DPC12 cellular apoptosis.

It has been reported that ROS formation, mitochondrial membrane depolarization, and ATP/ADP ratio decrement within mitochondria were noted in the brains of memory-impaired rats [28]. The neuroprotective effects of HE were investigated in AD mice, but due to the complex pathology and physiology in AD, it is difficult to develop an ideal animal model of AD for basic research [29]. A d-gal-induced neurodegenerative disease animal model was first reported by Chinese scholars [30]. Galactose alcohol, the metabolism of d-galactose, accumulates in cells, which results in the swelling and dysfunction of brain cells and, ultimately, nerve injuries [28]. Furthermore, excessive exposure to and accumulation of aluminum may be one cause of AD [31]. Aluminum causes neuronal vacuolization, followed by cerebral cortex atrophy and cognitive and memory dysfunction [31]. In AlCl_3_- and d-gal-induced AD mice, *Pleurotus ostreatus* polysaccharides successfully improved their cognition [32]. Learning and memory loss, slow behavior, and other signs of aging were widely noted in AD mice, and they were all strongly alleviated by HE based on autonomic activity analysis and rotarod and water maze tests.

Moreover, in the AD mice, HE significantly enhanced the Ach and ChAT concentrations in serum and the hypothalamus. In the brains of patients with AD, reduced ChAT activity and insufficient ACh content have been noted, as they drive the decline of learning and memory abilities [33]. This conclusion was supported by the evidence that *Flammulina velutipes* polysaccharides improve the scopolamine-induced impairment of learning and memory in rats by modulating Ach and ChAT levels [34]. As a major neurotransmitter regulating learning and memory, the observed enhancing activity of HE on ACh in our study may define an important pathway of cholinergic function during its neuroprotection in AD mice.

## 4. Materials and Methods

### 4.1. H. erinaceus Mycelium Aqueous Extract (HE) Preparation

*H. erinaceus* (CICC 14026; obtained from China Center of Industrial Culture Collection, Beijing, China) was cultured in a 100 L full-automatic fermenter (BaoXing Bioscience company, Shanghai, China) using a defined liquid medium as follows: 20 g/L of glucose, 20 g/L of tryptone, 3 g/L of KH_2_PO_4_, 2.0 g/L of MgSO_4_·7H_2_O, 0.1 g/L of vitamin B_1_. *H. erinaceus* mycelium was extracted twice in double distilled (D.D.) water at 80 °C for 3 h. After centrifuging at 5000 rpm for 15 min, HE was sequentially concentrated and freeze-dried for further experiments.

### 4.2. Cell Culture

PC12 cells (CRL-1721; ATCC; passages < 10) were cultured in Dulbecco’s modified eagle medium (DMEM) and supplemented with 10% fetal bovine serum (FBS), 5% horse serum (HS), penicillin (100 units/mL), and streptomycin (100 µg/mL), under a humidified atmosphere containing 5% CO_2_ at 37 °C. Except for morphology test, cells were exposed to 50 ng/mL of nerve growth factor (NGF; Sigma-Aldrich, St. Louis, MO, USA) dissolved in DMEM medium containing 1% FBS and HS for 48 h to differentiate. All agents applied in cell culture were purchased from Invitrogen, Carlsbad, CA, USA.

### 4.3. The Effect of HE on PC12 Cells Morphology

DPC12 cells were seeded into 6-well plate (2 × 10^4^ cells/well). After incubation, cells were exposed to HE (50 and 100 µg/mL) and NGF (50 ng/mL) for 24 and 48 h, and cell morphological changes were photographed by inverted microscope (10×; CKX31, Olympus, Tokyo, Japan).

### 4.4. Cell Viability Assessment

DPC12 cells were treated with 10, 25, 50, 100, 200, and 400 µg/mL of HE for 3 h, and then coincubated with or without 25 mM of l-Glu for another 24 h. MTT (3-(4,5-dimethyl-2-thiazolyl) 2,5-diphenyl-2*H*-tetrazolium bromide, Sigma-Aldrich) assay was applied for cell viability assessment, similar to previous research [14].

### 4.5. Nucleus Apoptosis Assessment

DPC12 cells were treated with 50 and 100 µg/mL of HE for 3 h, and then coincubated with 25 mM of l-Glu for another 24 h. Hoechst 33342 (Sigma-Aldrich) staining was performed to analyze the nucleus apoptosis similar to our previous study [14]. The changes of blue fluorescence intensity were determined by a fluorescent microscope (20×; CCD camera, TE2000, Nikon, Tokyo, Japan). The experiment was repeated six times. The relative fluorescence intensity was analyzed by Image J software (National Institutes of Health, Bethesda, Bethesda, MD, USA).

### 4.6. MMP Analysis

DPC12 cells were treated with HE (50 and 100 µg/mL) for 3 h, and then coincubated with 25 mM of l-Glu for another 12 h. According to previous research [14], JC-1 (Sigma-Aldrich) staining was performed to detect the changes in fluorescent color of mitochondria photographed by fluorescent microscope (20×; CCD camera, TE2000, Nikon, Tokyo, Japan). The experiment was repeated six times. The relative red and green fluorescence intensity was analyzed by Image J software (National Institutes of Health, Bethesda, MD, USA).

### 4.7. Intracellular Ca^2+^ Concentration Analysis

Similar as previous study [14], Fluo-4-AM (Invitrogen) staining was applied to analyze the intracellular Ca^2+^ concentration in DPC12 cells pretreated with 50 and 100 µg/mL of HE following with another 12-h coincubation with l-Glu. The green fluorescent intensity changes were detected by a fluorescent microscope (20×; CCD camera, TE2000, Nikon). The experiment was repeated six times. Green fluorescence intensity is on behalf of intracellular Ca^2+^ concentration. The relative fluorescence intensity was analyzed by Image J software (National Institutes of Health, Bethesda, MD, USA).

### 4.8. Intracellular ROS Levels Analysis

Intracellular ROS levels were detected by 2’,7’-dichlorofluorescein diacetate (DCFH-DA) (Nanjing Jiancheng Bioengineering Institute, Nanjing, China) staining [14]. DPC12 cells were treated with HE (50 and 100 µg/mL) for 3 h, and then coincubated with 25 mM of l-Glu for another 12 h. The green fluorescence intensity was detected with a fluorescent microscope (20×; CCD camera, TE2000, Nikon). The experiment was repeated six times. Green fluorescence intensity is on behalf of intracellular ROS accumulation. The relative fluorescence intensity was analyzed by Image J software (National Institutes of Health, Bethesda, MD, USA).

For ROS detection using flow cytometry assay, treated cells were collected and washed three times with phosphate-buffered saline (PBS), and then the intracellular ROS level was analyzed by flow cytometry (FC500, Beckman Coulter, Brea, CA, USA). The experiment was repeated three times.

### 4.9. Western Blot

DPC12 Cells were planted into 6-well plates at 4 × 10^5^ cells per well and treated with HE (50 and 100 µg/mL) and NGF (50 ng/mL) for 24 and 48 h. Cells were harvested and lysed with RIPA buffer (Sigma-Aldrich) containing 1% protease inhibitor cocktail (Sigma-Aldrich) and 2% PMSF (phenylmethanesulfonyl fluoride) (Sigma-Aldrich). After protein concentration detection by BCA Protein Assay Kit (Millipore, Billerica, MA, USA), 30 μg of proteins were separated using a 10% SDS-PAGE gel and transferred electrophoretically onto PVDF membranes. After blocking with 5% bull serum albumin (BSA) for 4 h, the transferred membranes were blotted with primary antibodies (β-tubulin III and glyceraldehyde-3-phosphate dehydrogenase (GAPDH)) (Abcam, Cambridge, MA, USA) at 4 °C overnight at a dilution of 1:1000, followed by incubation with horseradish peroxidase-conjugated secondary antibodies at a dilution of 1:2000 (Santa Cruz, CA, USA). Chemiluminescence was detected using ECL detection kits (Millipore) and imaging system (Biospectrun 600). The intensity of the bands was quantified by scanning densitometry using software Image J (National Institutes of Health, Bethesda, MD, USA).

### 4.10. Alzheimer's Disease Mouse Model Establishment and Drug Treatment Process

Experimental protocol was approved by the Institution Animal Ethics Committee of Jilin University (2015047; 27 February 2015). Balb/c female mice (20–22 g; 10 weeks) were housed in groups of 10 in clear plastic cages and maintained on a 12 h light/dark cycle (lights on 07:00–19:00 h) at 23 ± 1 °C with water and food available ad libitum.

**Figure 6:**
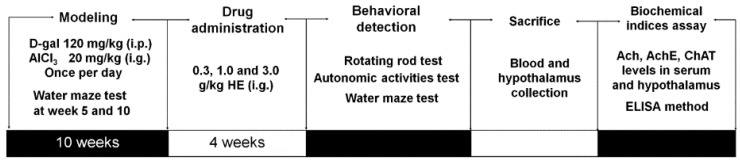
The experimental protocol for AlCl_3_- and d-gal-induced Alzheimer’s disease mouse model establishment and drug administration.

AD mouse model establishment and drug administration process is shown in Figure 6 [28]. 40 mice were subcutaneously injected with 120 mg/kg of d-gal and intragastrically administrated with 20 mg/kg of AlCl_3_ once per day continually for 10 weeks. Water maze tests were tested at the 5th and 10th week to evaluate modeling effect. All successfully developed AD mice were randomly divided for four groups (*n* = 10) and orally treated with normal saline (AD model mice), and HE at doses of 0.3, 1.0, and 3.0 g/kg for 4 weeks. Another 10 mice subcutaneously injected and orally treated with normal saline for 10 weeks, and intragastrically treated with normal saline for another 4 weeks served as control group. At the end of experiment, animal behavioral tests were assessed. Before sacrifice, blood was sampled under anesthesia. After sacrifice, hypothalamuses were collected from each mouse, and homogenized in D.D. water.

### 4.11. Behavioral Tests

#### 4.11.1. Autonomic Activities Test

Mice were placed into squares and their behaviors were observed for 5 min. Each square is in a dark state to detect the reaction ability of mice in darkness. Then the horizontal and vertical activities of each mouse were automatically recorded by the instrument.

#### 4.11.2. Fatigue Rotarod Test

Before formal testing, training was conducted three times at a speed on 20 rpm for 1 min. In the fatigue analysis, mice were placed on the turning device (ZB-200, Chengdu Techman Software Co., Ltd., Chengdu, China) with 20 rpm speed, and the time when mice fell off, induced by muscle fatigue, was recorded.

#### 4.11.3. Morris Water Maze Test

The instrument consists of a large circular pool and features four dead-end zones. Water with 10 cm depth and 25 ± 2 °C temperature was made opaque by addition of 1 L of milk, which prevented visualization of the platform. Five days before the test, mice were trained in a quiet environment with the same position of operator. The latency from immersion of mouse into the pool to escape onto the hidden platform was recorded. On the test day, mice were subjected to a 120 s probe trial in which the platform was obscure from the pool. The time spent within 120 s probe test time in target quadrant was recorded on an electronic time recorder.

### 4.12. Measurement of Ach and ChAT Levels in Serum and Hypothalamus

The levels of Ach and ChAT in serum and hypothalamus were detected by enzyme-linked immunosorbent assay (ELISA) according to the procedures provided by the related assay kits (Calbiotech, El Cajon, CA, USA).

### 4.13. Statistical Analysis

Data were expressed as mean ± SD. A one-way analysis of variance (ANOVA) was used to detect statistical significance followed by post hoc multiple comparisons (Dunn’s test). Statistical significance was accepted for *p* < 0.05.

## 5. Conclusions

In summary, HE protects DPC12 cells against l-Glu-induced neurotoxicity, mainly through mitochondria-related pathways. Further experiments performed in AlCl_3_- and d-gal-induced AD mice have confirmed HE’s protective effects, which may also involve the modulation of neurotransmitters. Our findings provide experimental evidence that HE may serve as a neuroprotective candidate for treating or preventing neurodegenerative diseases.

## Figures and Tables

**Table 1:** The effects of HE on bodyweights in Alzheimer’s disease mice.

Groups	Doses	28-Day Treatment				
		0	7	14	21	28
CTRL	–	23.5 ± 1.1	23.7 ± 1.1	24.0 ± 0.8	24.1 ± 0.9	24.2 ± 0.8
Model	–	23.9 ± 0.9	24.6 ± 1.1	25.0 ± 0.8	25.5 ± 0.5	25.3 ± 1.2
HE	0.3 g/kg	23.7 ± 0.7	24.0 ± 0.7	24.3 ± 0.8	24.5 ± 1.1	25.0 ± 0.8
HE	1 g/kg	23.9 ± 1.0	24.2 ± 0.6	24.4 ± 0.8	24.6 ± 1.0	25.0 ± 0.8
HE	3 g/kg	24.1 ± 0.8	24.3 ± 0.9	24.3 ± 0.8	24.8 ± 1.3	24.9 ± 0.9

Data are expressed as mean ± SD (*n* = 10) and analyzed by using a one-way ANOVA.

## Supplementary Materials

Supplementary materials can be found at www.mdpi.com/1422-0067/17/11/1810/s1.

## Abbreviations

ANOVA	A one-way analysis of variance
Ach	Acetylcholine
AD	Alzheimer’s disease
ChAT	Choline acetyltransferase
d-gal	d-Galactose
DPC12	Differentiated PC12
ELISA	Enzyme-linked immunosorbent assay
HE	*H. erinaceus* mycelium polysaccharides enriched aqueous extract
l-Glu	l-Glutamic acid
MMP	Mitochondrial membrane potential
NMDA	N Methyl D Aspartate
NGF	Nerve growth factor’s
PD	Parkinson’s disease
PC12 cell	Pheochromocytoma cell
ROS	Reactive oxygen species
