# Supplementary material for: The Neuroprotective Properties of Hericium erinaceus in Glutamate-Damaged Differentiated PC12 Cells and an Alzheimer’s Disease Mouse Model
Source: Int J Mol Sci. 2016 Nov 1;17(11):1810. doi: 10.3390/ijms17111810 (PMC5133811; doi:10.3390/ijms17111810)
Supplement: Supplementary file 1 [file ijms-17-01810-s001.pdf]

Supplementary Materials: The Neuroprotective Properties of *Hericium erinaceus* in Glutamate-Damaged Differentiated PC12 Cells and an Alzheimer's Disease Mouse Model

Junrong Zhang, Shengshu An, Wenji Hu, Meiyu Teng, Xue Wang, Yidi Qu, Yang Liu, Ye Yuan and Di Wang

2. Results

The Effects of HE on L-Glu-Induced Intracellular ROS Accumulation

Three-hour HE preincubation followed by another 12 h coexposure to L-Glu strongly reduced high green fluorescence in DCFH-DA staining, suggesting its inhibition of ROS accumulation (Figure S1).

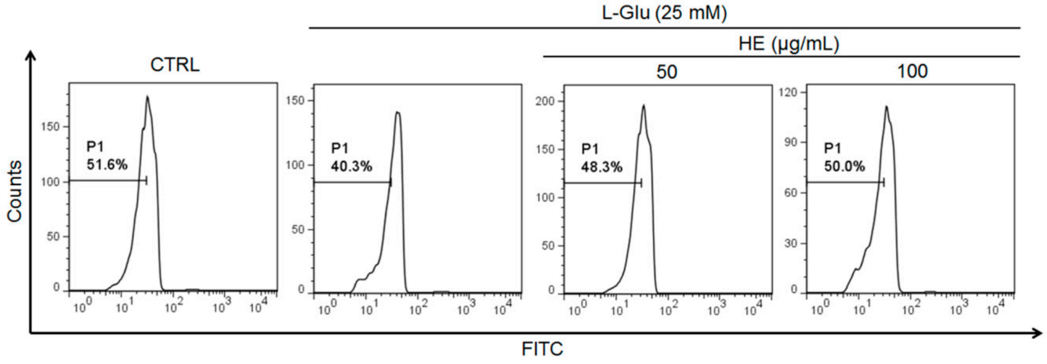

Figure S1. The overaccumulation of ROS caused by 12 h L-Glu incubation was significantly reduced by 3 h HE pretreatment, detected via DCFH-DA staining ($n = 3$).

4. Methods and Materials

Assessment of ROS

The intracellular ROS level was measured by 2′,7′-Dichlorodihydrofluorescein diacetate (DCFH-DA, Sigma-Aldrich, St. Louis, Missouri, USA) staining. Cells were seeded into 6-well plates at 2×10^5 cells per well. DPC12 cells were treated with HE (50 and 100 μg/mL) for 3 h, and then coincubated with 25 mM of L-Glu for another 12 h. After three washes with phosphate-buffered saline (PBS), the changes of intracellular ROS level were analyzed by flow cytometry (FC500, Beckman Coulter, Brea, CA, USA). The experiment was repeated three times.
